# Flight and Dietary Antioxidants Influence Antioxidant Expression and Activity in a Migratory Bird

**DOI:** 10.1093/iob/obab035

**Published:** 2021-12-30

**Authors:** Kristen J DeMoranville, Wales A Carter, Barbara J Pierce, Scott R McWilliams

**Affiliations:** Department of Natural Resources Science, University of Rhode Island, 102 Coastal Institute, 1 Greenhouse Road, Kingstown, RI 02881, USA; Department of Natural Resources Science, University of Rhode Island, 102 Coastal Institute, 1 Greenhouse Road, Kingstown, RI 02881, USA; Department of Biology, Sacred Heart University, 5151 Park Ave, Fairfield, CT 06825, USA; Department of Natural Resources Science, University of Rhode Island, 102 Coastal Institute, 1 Greenhouse Road, Kingstown, RI 02881, USA

## Abstract

Ecologically relevant factors such as exercise and diet quality can directly influence how physiological systems work including those involved in maintaining oxidative balance; however, to our knowledge, no studies to date have focused on how such factors directly affect expression of key components of the endogenous antioxidant system (i.e., transcription factors, select antioxidant genes, and corresponding antioxidant enzymes) in several metabolically active tissues of a migratory songbird. We conducted a three-factor experiment that tested the following hypotheses: (H1) Daily flying over several weeks increases the expression of transcription factors NRF2 and PPARs as well as endogenous antioxidant genes (i.e., CAT, SOD1, SOD2, GPX1, GPX4), and upregulates endogenous antioxidant enzyme activities (i.e., CAT, SOD, GPx). (H2) Songbirds fed diets composed of more 18:2n-6 PUFA are more susceptible to oxidative damage and thus upregulate their endogenous antioxidant system compared with when fed diets with less PUFA. (H3) Songbirds fed dietary anthocyanins gain additional antioxidant protection and thus upregulate their endogenous antioxidant system less compared with songbirds not fed anthocyanins. Flight training increased the expression of 3 of the 6 antioxidant genes and transcription factors measured in the liver, consistent with H1, but for only one gene (SOD2) in the pectoralis. Dietary fat quality had no effect on antioxidant pathways (H2), whereas dietary anthocyanins increased the expression of select antioxidant enzymes in the pectoralis, but not in the liver (H3). These tissue-specific differences in response to flying and dietary antioxidants are likely explained by functional differences between tissues as well as fundamental differences in their turnover rates. The consumption of dietary antioxidants along with regular flying enables birds during migration to stimulate the expression of genes involved in antioxidant protection likely through increasing the transcriptional activity of NRF2 and PPARs, and thereby demonstrates for the first time that these relevant ecological factors affect the regulation of key antioxidant pathways in wild birds. What remains to be demonstrated is how the extent of these ecological factors (i.e., intensity or duration of flight, amounts of dietary antioxidants) influences the regulation of these antioxidant pathways and thus oxidative balance.

## German abstract

Ökologisch relevante Faktoren, wie energetisch aufwändige Bewegungsabläufe oder sich verändernde Ernährungsbedingungen, können direkten Einfluss auf physiologische Prozesse im Körper haben, und somit auch Systeme beeinflussen, die die Sauerstoffbalance involviert sind.

Allerdings ist nicht viel darüber bekannt, wie sich die oben genannten Faktoren direkt auf die Expression von Schlüsselkomponenten (z.B. Transkriptionsfaktoren, antioxidative Gene und entsprechende antioxidative Enzyme) körpereigener antioxidativer Systeme in metabolisch aktiven Geweben einer ziehenden Singvogelart auswirken.

Wir führten ein 3-faktorielles Experiment durch, in dem wir die folgenden Hypothesen testeten:

(H1) Ein tägliches Flugtraining über einige Wochen führt zu einer Steigerung der Expression von körpereigenen Transkriptionsfaktoren (NRF2 und PPARs) und antioxidativen Genen (z.B. CAT, SOD1, SOD2, GPX1, GPX4) und erhöht die Aktivität endogener, antioxidativer Enzyme (z.B. CAT, SOD, GPx).

(H2) Singvögel, denen eine Diät aus vermehrt 18:2n-6 mehrfach ungesättigten Fettsäuren (PUFA) verfüttert wird, haben im Gegensatz zu einer Diät mit geringem PUFA Anteil, eine erhöhte Anfälligkeit für oxidative Schäden und werden ihr körpereigenes, antioxidatives System verstärkt aktivieren.

(H3) Singvögel, die mit einer Diät reich an antioxidativen Anthocyanen gefüttert werden, sind weniger anfällig für oxidative Schäden und werden ihr körpereigenes, antioxidatives System weniger hochregulieren, als bei einer Diät ohne Anthocyane.

Resultate unserer Studien zeigen, dass Flugtraining eine verstärkte Expression von drei der sechs untersuchten antioxidativen Genen in der Leber bewirkt. Ebenfalls erhöht im Lebergewebe waren die Werte der Transkriptionsfaktoren (H1), während im Brustmuskel nur ein antioxidatives Gen erhöht exprimiert wurde.

Qualität und Zusammensetzung der Fettsäuren in der Diät hatten keinen Effekt auf die antioxidativen Prozesse (H2), während diätisch verabreichte Anthocyane die Expression von speziell antioxidativen Enzymen im Brustmuskel, aber nicht in der Leber bewirkten (H3).

Diese gewebespezifischen Unterschiede auf Grund von Flugtraining und diätischen Anthocyanen, können durch funktionelle, ebenso wie umsatzbedingte Unterschiede erklärt werden. Die Aufnahme von Anthocyanen vor und während des Zugs ermöglicht es den Vögeln gezielt die Expression von Genen zu stimulieren, die zum antioxidativen Schutz beitragen. Dies geschieht höchstwahrscheinlich durch die transkriptionelle Aktivierung von NRF2 und PPARs. Mit diesen Ergebnissen lässt sich zum ersten Mal zeigen, dass sich ökologischen Faktoren direkt auf die Regulation von wichtigen, körpereigenen Prozessen auswirken und eine antioxidative Schutzmaßnahme in Vögeln hervorrufen. Es bleibt herauszufinden, wie sehr sich das Ausmaß der ökologischen Faktoren (Dauer und Intensität des Fluges, Dosierung der Anthocyane) auf die Regulierung antioxidativer Prozesse auswirkt und somit die endogene oxidative Balance beeinflusst.

## Introduction

### The challenges of oxidative balance for wild vertebrates, and specifically migratory birds

Aerobically respiring organisms must maintain a balanced oxidative status where excess reactive species are neutralized with antioxidants to minimize resulting oxidative damage or such damage must be repaired (Halliwell and Gutteridge 2007; Powers and Jackson 2008; Costantini 2019). Maintaining oxidative balance is especially crucial when reactive species production is high during energetically demanding life history stages including migration (Jenni-Eiermann et al. 2014; Skrip et al. 2015; Eikenaar et al. 2017; Gutiérrez et al. 2019) and reproduction (Wiersma et al. 2004; Costantini et al. 2010, 2020; Speakman and Garratt 2013; Mentesana et al. 2018). Migratory birds are particularly vulnerable to oxidative damage since they must increase their metabolism nine times above their basal metabolic rates to complete energy-intense long-distance flights (Swanson 2010; Corder and Schaeffer 2015; Butler 2016; DeMoranville et al. 2019). However, like other vertebrates, migratory birds can avoid or ameliorate the production of reactive species using endogenously produced antioxidant enzymes (e.g., catalase [CAT]), sacrificial molecules (e.g., uric acid), or dietary antioxidants (e.g., anthocyanins) to minimize oxidative damage (Halliwell and Gutteridge 2007; Jenni-Eiermann et al. 2014; Cooper-Mullin and McWilliams 2016; Skrip and McWilliams 2016). This multifaceted antioxidant system has been typically investigated by measuring the final products of antioxidants and oxidative damage (e.g., enzyme activities, nonenzymatic antioxidant capacity, lipid peroxidation capacity, protein carbonyls), and mixed results suggest that sometimes migratory birds can maintain oxidative balance when exercising (Cooper-Mullin et al. 2019) but sometimes cannot (Skrip et al. 2016; Dick and Guglielmo 2019). Conflicting results may be due to indirect effects of environmental stimuli (e.g., exercise, diet) on measures of biochemical antioxidant capacity and oxidative damage levels since these measures depend on many factors including transcriptional and posttranscriptional regulation. In contrast, environmental factors such as exercise and diet can directly influence antioxidant molecular pathways; thus, studies that quantify the gene expression of antioxidant transcription factors and endogenous antioxidants in response to environmental factors can better elucidate how this multifaceted antioxidant system is regulated (Costantini 2019).

#### The antioxidant response of animals within the context of ecology

##### The antioxidant response begins with transcription factors

The major cellular pathways in all animals for regulating the antioxidant response include peroxisome proliferator-activated nuclear receptors (PPARs) and nuclear factor erythroid 2-related factor 2 (NRF2). These transcription factors regulate endogenous antioxidant enzymes, and both transcription factors can be affected by ecologically relevant factors including exercise (Spangenburg et al. 2009; Thomas et al. 2012; Done and Traustadóttir 2016; Done et al. 2017) and diet (Gomez-Cabrera et al. 2008; Ristow et al. 2009; Pierce and McWilliams 2014; Tian et al. 2019). There are three PPAR isoforms, PPARγ, PPARδ, and PPARα, that act as key regulators of fat metabolism (Bensinger and Tontonoz 2008; Ehrenborg and Krook 2009; Wang 2010) and the production of reactive oxygen species (Zhang et al. 2015) and so may be important for birds that rely primarily on fat for fuel and must contend with the production of lipid peroxides (Cooper-Mullin and McWilliams 2016; Skrip and McWilliams 2016). The stimulation of PPAR pathways increases fat metabolism and oxidation, and thus may induce reactive species production and cause an imbalanced oxidative status (Kim and Yang 2013). However, PPARs can also protect against reactive species by directly transcribing superoxide dismutase (SOD), glutathione peroxidase (GPx), and CAT through PPAR response element (PPRE) sequences located in each enzyme's promoter regions (Kim and Yang 2013). To our knowledge, the PPAR regulation of antioxidant enzymes has not been examined in songbirds or other wildlife.

NRF2 binds to the antioxidant response element (ARE) to transcribe an array of 250 genes involved in antioxidant protection and redox homeostasis (Tebay et al. 2015; Yamamoto et al. 2018) including SOD (Park and Rho 2002; Dreger et al. 2009) and glutathione, the cofactor to GPx (Tebay et al. 2015; Yamamoto et al. 2018), whereas CAT activity is induced by NRF2 without an apparent ARE promoter region (Venugopal and Jaiswal 1998; Zhu et al. 2005, 2008). In most taxa NRF2 is regulated by a Kelch-like ECH associated protein 1 (KEAP1) repressor that has recently been shown to have mutated in songbirds and their relatives (i.e., Neoaves) and resulted in a constituently active NRF2 that is able to transcribe antioxidant genes under any cellular conditions (Lewis et al. 2010; Castiglione et al. 2020). A constituently active NRF2 would allow birds to quickly transcribe genes associated with antioxidant enzymes during times of high reactive species production, like during migratory flight. Studying molecular antioxidant pathways such as those described earlier in songbirds is particularly interesting due to the novel continuous activation of NRF2 recently discovered in birds, their energy-expensive lifestyle and mode of locomotion (i.e., flying is costly), and the potential importance of ecologically relevant factors such as exercise and diet quality on the functioning of these antioxidant pathways.

##### Exercise stimulates specific molecular antioxidant pathways

Exercise and the associated increases in metabolism can stimulate molecular antioxidant pathways by (1) increasing the production of reactive species resulting in increased NRF2 transcription (Done and Traustadóttir 2016) and (2) increasing the amount of circulating fatty acid ligands (e.g., free fatty acids, eicosanoids) resulting in increased PPAR coactivator activity and PPAR signaling (Baar et al. 2002; Thomas et al. 2012). The NRF2 antioxidant pathway in muscle is stimulated by both acute exercise and exercise training in mice and humans (Done and Traustadóttir 2016; Wang et al. 2016; Done et al. 2017). Repeated bouts of exercise have a similar stimulatory effect on NRF2 pathways in multiple tissues including skeletal muscle, myocardium, liver, kidney, brain, testes, and prostate (Done and Traustadóttir 2016). NRF2 pathways are required for increases in endurance performance and antioxidant protection associated with exercise training as indicated by the inability of NRF2-deficient mice to increase NRF2 mRNA levels, mitochondrial biogenesis, and SOD and CAT expression after 5 weeks of training on a treadmill compared with wild-type mice (Merry and Ristow 2016). The stimulatory effect of exercise on NRF2 has not been studied in songbirds, although it is clearly relevant given their need to contend with oxidative challenges associated with regularly flying.

Exercise also stimulates PPAR pathways mainly through the generation of PPAR fat ligands and increased expression of peroxisome proliferator-activated receptor gamma coactivator (PGC-1) cofactors that bind to PPARs to increase their transcriptional activity (Baar et al. 2002; Finck and Kelly 2006; Thomas et al. 2012). An 8-week cycling training regime demonstrated that exercise training increased plasma PPAR ligands, PPARγ activity, and PPAR target gene expression (CD36, LXRα, ABCA1) within 3 h postexercise (Thomas et al. 2012). Similarly, the PGC-1 coactivators increased two-fold within 18 h of a single bout of swimming exercise in rats suggesting a possible increase in PPAR activity (Baar et al. 2002). When PPARs, cofactors, and antioxidant enzymes were studied simultaneously, exercise-induced ROS production increased the mRNA expression of PGC-1α, PGC-1β, PPARγ, SOD1, SOD2, GPX1, and CAT in human skeletal muscle (Ristow et al. 2009). No previous studies have simultaneously investigated how exercise influences PPAR expression and antioxidant enzyme expression or activity in wild birds to determine whether this pathway is important in protecting against exercise-induced reactive species during migration.

##### Dietary fat challenges the endogenous antioxidant system

Birds rely on fatty acids to fuel flight (McWilliams et al. 2004; Guglielmo 2018), and certain migratory songbird species optimize the relative amounts of polyunsaturated fat (PUFA) to monounsaturated fat (MUFA) in their diets, fat stores, and in circulation (Pierce et al. 2004; Pierce and McWilliams 2005; Price et al. 2008; Smith and McWilliams 2010). The potential benefits of consuming 18:2n-6 PUFA (linoleic acid) include faster mobilization rates, maintaining optimal membrane properties, and increases in PPAR activation (Pierce and McWilliams 2014; Guglielmo 2018). However, all PUFA are highly susceptible to oxidative damage due to easily oxidizable hydrogen atoms located near their double bonds (Wagner et al. 1994; Niki et al. 2005). Furthermore, lipid radicals are produced when reactive species scavenge the hydrogen atoms from an unsaturated fat, and often this causes a self-perpetuating chain reaction damaging nearby PUFAs and other molecules (Wagner et al. 1994; Niki et al. 2005). Thus, there is a potential trade-off of using 18:2n-6 PUFA as substrate to enhance metabolism versus battling its associated oxidative costs (McWilliams et al. 2020) that may require more endogenous and dietary antioxidant protection.

##### Dietary antioxidants modulate molecular antioxidant pathways:

Many songbird species select fruits high in antioxidants during migration, suggesting that antioxidant consumption is important to protect against oxidative damage during this life history stage (Alan et al. 2013; Bolser et al. 2013). Water-soluble antioxidants such as anthocyanins are particularly relevant in songbirds since they are preferentially consumed by certain species during fall migration and in captivity (Schaefer et al. 2008; Alan et al. 2013; Bolser et al. 2013). Dietary anthocyanin supplements in humans stimulate NRF2 and enhance antioxidant capacity in the context of the inflammatory disease atherosclerosis (Aboonabi and Singh 2015), in human diabetic aortic cells (Aboonabi et al. 2020), cloned rat liver cells (Shih et al. 2007), human serum under mild hypoxic conditions (Cimino et al. 2013), and in healthy dairy goats (Tian et al. 2019). This “stimulatory” hypothesis identifies dietary anthocyanins as enhancers of the NRF2 antioxidant pathway, yet the exact mechanisms responsible remain unclear (Shih et al. 2007; Cimino et al. 2013) and have not yet been studied in birds. Interestingly, when dietary antioxidant supplements (e.g., vitamins C and E) are combined with exercise in mice and humans, reactive species production is reduced, there is a decrease in the transcription of NRF2 (Done and Traustadóttir 2016), and a decreased activation of PPAR pathways (Gomez-Cabrera et al. 2008; Ristow et al. 2009). This is a potentially energetically beneficial strategy since organisms can use antioxidants gained through their diet for reactive species protection to avoid the energetically expensive production and maintenance of endogenous antioxidant enzymes. This “compensatory” hypothesis may only be relevant when individuals are metabolically challenged, although this hypothesis has not been tested in any nonhuman model system. Considered together, these studies suggest that birds consuming antioxidant-rich berries may either use dietary anthocyanins to (1) stimulate NRF2 transcription of antioxidant enzymes and/or (2) to quench reactive species and inhibit the energetically costly transcription of antioxidant enzymes. These scenarios may not be exclusive, but rather depend on current oxidative status and energetic demands.

### How do flight training, dietary fat, and dietary anthocyanins affect the endogenous antioxidant system?

The goal of this experimental study was to investigate how flight training in a wind tunnel as well as consumption of certain dietary fats (i.e., 18:2n-6 PUFA) and dietary antioxidants (i.e., water-soluble anthocyanins) affected the expression of NRF2 and PPAR transcription factors, select antioxidant genes, and corresponding antioxidant enzymes in the liver and the pectoralis muscle of a migratory songbird. We tested the following three hypotheses: Flight training effect (H1): flying regularly over several weeks (1) increases the expression of NRF2 and PPARs, and thereby (2) increases the expression of endogenous antioxidant genes (i.e. CAT, SOD1, SOD2, GPX1, GPX4), and (3) produces a coordinated upregulation of endogenous antioxidant enzyme activities (i.e. CAT, SOD, GPx). Dietary fat effect (H2): migratory songbirds fed diets composed of more PUFA are more susceptible to oxidative damage and thus have increased expression levels of NRF2 and PPAR transcription factors, selected antioxidant genes, and corresponding antioxidant enzyme activities compared with birds fed diets with less PUFA. Dietary antioxidant effect (H3): migratory songbirds fed dietary anthocyanins have less need to upregulate their endogenous antioxidant system and thus have decreased expression levels of the NRF2 and PPAR transcription factors, selected antioxidant genes, and corresponding antioxidant enzymes compared with songbirds not fed anthocyanins. We also examined whether these three ecologically relevant factors (flying, fat quality of diet, dietary antioxidants) significantly interacted to affect key components of the antioxidant system. For example, the compensatory function of dietary antioxidants may be most evident in birds that are flight trained due to their inhibitory effect on NRF2 and PPARs and the transcription of antioxidant enzymes. Whereas, a stimulatory function of dietary antioxidants may be evident in untrained birds due to their stimulatory effects on NRF2 activity in organisms at rest.

## Materials and methods

### Experimental design

Omnivorous migratory songbirds undergo endurance flights biannually and many species switch to eating mostly berries that are rich in fats and antioxidants during their fall migration (Alan et al. 2013; Bolser et al. 2013); thus, they are an ideal natural system to study how the endogenous antioxidant system responds to flight training, dietary antioxidants, and dietary fat. We used European Starlings (*Sturnus vulgaris*) as representative songbirds for this study because they are abundant in the New World and Old World, they are omnivorous and acclimate well to captivity and new diets, and they have been successfully trained and flown in wind tunnels in other studies (Hall et al. 2014; Casagrande et al. 2020). Hatch year European starlings were caught at a dairy farm 20 km north of the Advanced Facility for Avian Research (AFAR), University of Western Ontario, London, Ontario, before fall migration, between 19 and 23 August 2015. Starlings from this southern Canada wild population are considered partial migrants that fly short distances as inferred by banding records (Dolbeer 1982; Cabe 1993). Therefore, flights ranging from 1 to 3 h may constitute as intense exercise for the starlings in our experiment. Starlings were housed in one of four large indoor aviaries at AFAR (two 2.4 m × 3.7 m × 3.1 m and two 2.4 m × 2.3 m × 3.5 m). On August 24th we measured morphological characteristics, body mass, and molt score (0–5; Ginn and Melville 1983) for each individual. Birds were then randomly sorted into four groups with roughly equal distributions of body size and molt score. We maintained aviaries at 21°C on a natural light cycle from capture and until the start of the experiment on September 21st when we fixed the light schedule at 11:13 L:D (day length on this date in London, Ontario). We did not directly verify that such typical fall decreases in light levels increased food intake in starlings, although many studies provide such evidence in migratory birds (Gwinner 1996; Helm et al. 2009; Bulte and Bairlein 2013), or to increase Zugunruhe since starlings are diurnal migrants. Upon capture and until the start of the experiment each week we weighed and inspected all birds to assess their health. All birds were cared for under animal care protocols for University of Western Ontario (2010-216) and the University of Rhode Island (AN11-12-009).

### Experimental diets

Birds had *ad libitum* access to one of two semi-synthetic diets that had the same macronutrient content as a lipid-rich fruit diet (41% carbohydrate, 13% protein, 30% fat) and differed only in fatty acid composition. We manipulated the proportions of canola, sunflower, and palm oil so that the diets were either high (32%) or low (13%) in 18:2n-6 PUFA (linoleic acid) that was primarily traded off with 16:0 (palmitic acid). Thus, our experimental design requires us to attribute any observed dietary fat effects to both 18:2n-6 and 16:0 content. However, our interpretations focus on the potential effects of 18:2n-6 due to its demonstrated importance in metabolic signaling (Forman et al. 1997; Kennedy et al. 2007; Hamilton et al. 2018; Dick and Guglielmo 2019). The complete list of diet ingredients and amounts have been previously published (Carter et al. 2020). Starlings in two aviaries received a 13% 18:2n-6 diet and two others received a 32% 18:2n-6 diet. The two diets have been shown to produce reliable differences in tissue fatty acid composition of starlings (Carter et al. 2020). On September 1, we began adding a supplementary water-soluble antioxidant, anthocyanin (elderberry powder; Artemis International, Fort Wayne, IN) to the diets of one 13% 18:2n-6 aviary and one 32% 18:2n-6 aviary, producing a 2 × 2 factorial diet manipulation with four diet groups: 13% 18:2n-6, anthocyanin unsupplemented (*N* = 23); 13% 18:2n-6, anthocyanin supplemented (*N* = 23); 32% 18:2n-6, anthocyanin unsupplemented (*N* = 21); and 32% 18:2n-6, anthocyanin supplemented (*N* = 20). We chose the anthocyanin concentration used by researchers studying the effects of anthocyanin supplementation on food choice and immunocompetence in European blackcaps, *Sylvia atricapilla* (Catoni et al. 2008; Schaefer et al. 2008). The anthocyanin supplement was equal to eating 2.8 mg per day that is equal to consuming 17 berries per day based on an average daily synthetic diet consumption of 35 wet g day^−1^ (as observed in food intake trials in this study). Anthocyanins are particularly relevant to songbirds since they are prevalent in fruits consumed during migration (Catoni et al. 2008; Schaefer et al. 2008; Alan et al. 2013; Bolser et al. 2013), and anthocyanins are exclusively stable at acidic pH levels and are more likely to be preserved for utilization in the 2× more acidic stomachs of songbirds (pH 2) relative to mammals (pH 4.4) (Dangles and Fenger 2018).

### Experimental timeline

**Fig. 1:**
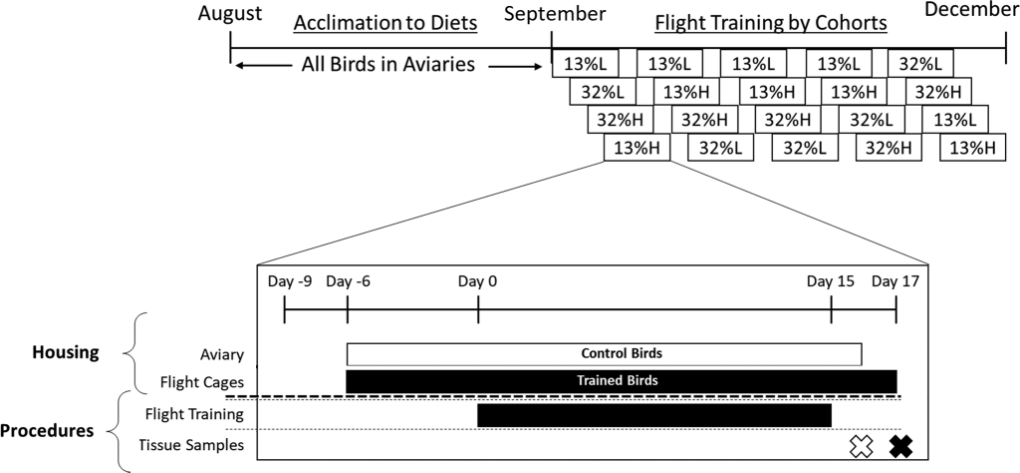
The experimental timeline for the experiment (modified from Carter et al. 2020) conducted between August and December 2015. (Top timeline) Starlings were captured during early August and initially housed in one of four large indoor aviaries where they had *ad libitum* access to one of two semi-synthetic diets that had the same macronutrient content as common lipid-rich fruits (41% carbohydrate, 13% protein, 30% fat) with a high (32%) or low (13%) 18:2n-6 content and with (“H” for high) or without (“L” for low) a water-soluble antioxidant, anthocyanin. Prior to flight training, we randomly assigned five starlings (two untrained, three destined to be flight trained) to each of 20 cohorts. There were five cohorts per diet group, and the sampling order of the diet groups was randomly assigned within a cohort group ensuring that the same diet group was not consistently sampled first or last, and all diet groups were sampled within 10 days of one another. (Bottom expanded timeline for each cohort) On September 23rd, and continuing every 3 days thereafter, the three flight-trained individuals from each selected cohort were moved from their large aviaries to smaller, mobile cages (see text for details) while the two untrained birds from this cohort remained in their large aviary. Flight-trained birds were acclimated to the flight cages and the wind tunnel for 6 days and then 15 days of flight training in the wind tunnel (see text for details). For logistical reasons, untrained and flight-trained birds were sacrificed, and the liver and pectoralis muscle sampled at 1400–1500 h on days 16 and 17, respectively (see text for details).

On September 21st we randomly assigned five starlings to each of 20 cohorts. There were five cohorts per diet group, and the sampling order of the diet groups was randomly assigned within a cohort group ensuring that the same diet group was not consistently sampled first or last, and all diet groups were sampled within 10 days of one another. On September 23rd, and continuing every 3 days thereafter (Fig. 1), the five individuals from each selected cohort were removed from their aviaries, and we randomly assigned two birds as untrained birds and three birds as flight-trained birds. Each selected cohort was placed in individual cages (0.6 m × 0.5 m × 0.5 m) for 2 days (days 9 and 8 relative to flight training) to measure food intake and another 2 days (days 7 and 5) to measure basal and peak metabolic rates (Carter et al. 2020). On day 5 we returned the two untrained birds to their original aviary and moved the three flight-trained birds to a 0.8 m × 1.5 m × 2 m flight aviary.

### Flight training

In order to assess the impact of diet and endurance flight on the endogenous antioxidant system, three flight-trained birds were flown in a wind tunnel for 4 days of pretraining followed by 15 days of flight training. Such a flight training regime has demonstrated success at eliciting long-duration flights in starlings (Engel et al. 2006). The wind tunnel was set to 12 m/s wind speed, 15°C, and 70% humidity, and birds were fasted for 1 h prior to all flights. Pretraining (days 4 to 1) included training birds to fly between their flight cage and the wind tunnel and 20 min of habituation time per day in the wind tunnel with a perch. These initial four “pretraining” days were not included in the reported overall training time. Flight-trained starlings then participated in a 15-day training regimen that consisted of increasing periods of flight (20–180 min) in the wind tunnel as follows: days 1–4, 20 min each day; days 5–6, 30 min each day; day 7, 60 min; day 8, 90 min; day 9, 30 min; day 10, 120 min; day 11, 180 min; day 12, rest day; day 13, 60 min; and day 14, 30 min. This flight training culminated in a flight on day 15 that lasted as long as birds would voluntarily fly, up to 6 h. The final flight was on average 193 min ± 71 and the maximum was 360 min. At 1400–1500 h on days 16 and 17 the untrained and trained birds, respectively, in each cohort were euthanized by cervical dislocation while under isoflurane anesthesia, and the liver and pectoralis muscle samples were collected and immediately weighed. All tissues were flash frozen in liquid nitrogen and stored at −80°C until analysis. This sampling design allowed us to compare gene expression and enzyme activities in the liver and pectoralis of untrained (control) birds and flight-trained birds that had recovered (for 48 h) from their longest flight on day 15.

### Quantitative reverse transcription PCR

Quantitative reverse transcription PCR (qRT-polymerase chain reaction (PCR)) was performed to quantify relative expression of select antioxidant genes, NRF2, and PPARs. Total RNA was extracted from liver and pectoralis muscle (25–30 mg) using RNeasy® Fibrous Mini Kit (QIAGEN^®^, Germantown, MD, USA) following kit instructions including the recommended DNase treatment step, but without the proteinase K digestion step for the liver. RNA concentrations and quality were verified using a NanoDrop (NanoDrop Technologies, Wilmington, DE, USA). RNA (0.5 μg) was reverse transcribed using the SuperScript IV First-strand Synthesis System Kit (Thermofisher, Burlington, ON, CA), and cDNA was used as a template for qPCR. Each 17.5 μL PCR reaction mixture was comprised of 1:15 diluted cDNA template, 400 nM gene-specific primers, and The Applied Biosystems™ PowerUp™ SYBR™ Green Master Mix (Thermofisher, Burlington, ON, CA). The temperature cycles for each PCR reaction were as follows: 2 min at 50°C, 2 min at 95°C, 45 cycles of 95°C for 15 s, and a primer-specific optimal temperature (62–68°C) for 1 min. Each PCR run was completed with a melt curve analysis to confirm the presence of a single PCR product, and amplification efficiency was verified for every primer pair. The gene expression values were derived from a standard curve generated for each primer set. Primer sequences were derived using NCBI's BLASTN v2.10.0 program and searching the European starling's genome database (Sturnus_vulgaris-1.0 reference Annotation Release 100) for predicted genes. Primers were designed so that at least one primer was exon spanning. Primers in our study met the following criteria: amplification of a single product indicated by a single peak in the melting curve analysis and efficiency of amplification between 98% and 100%. In all cases, cycle threshold (Ct) values ranged from 18 to 29, except for PPARγ that was detected between 30 and 32 range. Primer sequences and GenBank accession numbers are shown in Table S1.

Transcript expression levels were normalized to the reference gene β-actin, which codes for the β-actin gene responsible for the structure and motility of cells, and is highly conserved across tissues and avian species (Bernard et al. 1999; Mcfarlan et al. 2009; Perfito et al. 2015; Cordes et al. 2016; Zhang et al. 2018). β-Actin did not vary across the eight diet and training treatments in the pectoralis (F_8,80_ = 1.68, *P* = 0.12) or liver (F_8,78_ = 0.94, *P* = 0.49) or over the course of the experiment in the pectoralis (Julian date: estimate ± standard error 0.02 ± 0.02, *P* = 0.30) or liver (Julian date: estimate ± standard error 0.01 ± 0.02, *P* = 0.67). Transcript expression normalized to β-actin was used for causal pathway analyses. Normalized transcript expression relative to the 13% 18:2n-6, low antioxidant, untrained reference group was used for all linear models, a quantification method referred to as relative gene expression.

### Antioxidant enzyme activities

In preparation for the measurement of antioxidant enzyme activity, approximately 250 mg of tissue was homogenized on ice in nine volumes of 0.1 M phosphate buffer, pH 7 with 3 × 10 s pulses of a high-speed stainless-steel homogenizer (Tissue Master 125, Omni International, Kennesaw, GA, USA). Homogenate was centrifuged at 10,000 *g* for 10 min at 4°C (Beckman Coulter Allegra 21R, Indianapolis, IN, USA), and the supernatant was aliquoted to three separate tubes (∼200 μL per tube) to conduct the four separate assays (Bradford, CAT, SOD, GPx). A chelating agent (EDTA) was added to the tubes used to measure enzymatic antioxidant activity to protect the sample from the rapid autoxidation from trace metal ions within the sample, resulting in a final buffer containing 0.05 M phosphate-buffered saline and 0.1 mM EDTA, pH 7. Supernatant was immediately frozen at −80°C until the time of the assay (1–3 months after homogenization).

The activities of CAT, SOD, and GPx enzymes were assayed according to Cayman Chemical (Cayman Chemical Company, Ann Arbor, Michigan, USA) commercial kit protocols (Catalase Assay Kit 707002, Superoxide Dismutase Assay Kit 706002, Glutathione Peroxidase Assay Kit 703102), and all enzyme activities were normalized to soluble protein content (mg/mL) as measured by the Bradford protein assay (Biorad, 5000006) using a bovine albumin serum standard (Fisher Scientific AAJ6477709) (Dick and Guglielmo 2019). All assays were conducted on a microplate and read in a plate reader (BioTek Synergy HTX, Winooski, Vermont, USA) in duplicate or triplicate, until the %CVs among replicates were under 13%. The final dilution factors for the assays of the pectoralis were: Bradford 1:200, CAT 1:10, SOD 1:100, GPx 1:10, and in the liver: Bradford 1:1000, CAT 1:250, SOD 1:100, and GPx 1:50. CAT catalyzes the oxidation of aliphatic alcohol, which acts as an electron donor for hydroperoxides, and the assay measured the amount of oxidized aldehydes present after termination of the reaction (nmol/min/mL) (Jimenez et al. 2020a, 2020b). The SOD assay measured all three types of SOD (Cu/Zn, Mn, and FeSOD) present by detecting the amount of superoxide radicals generated by xanthine oxidase and hypoxanthine using tetrazolium salt for detection (concentration unit = U/mL, one unit is defined as the amount of enzyme needed to exhibit 50% dismutation of the superoxide radical) (Dick and Guglielmo 2019). GPx activity of all present GPx types (GPx 1–5) was measured indirectly, as oxidized glutathione produced upon reduction of hydroperoxides by GPx is recycled to its reduced state by glutathione reductase and NADPH, and the resulting rate of decrease is directly proportional to GPx activity (nmol/min/mL) (Cooper-Mullin et al. 2019).

### Statistics

#### Linear models

We used R (v3.5.3; R Core Team 2019, Vienna, Austria) for all analyses. Linear models were constructed to test the hypothesis that flight training (H1), dietary fat (H2), and dietary antioxidants (H3) influenced the gene expression of NRF2 and PPAR transcription factors, their antioxidant genes, and corresponding antioxidant enzyme activities. We used a global model without interaction terms that best matched this hypothesis and included possible explanatory covariates (i.e., Julian date, sex, and wing chord). Nonsignificant explanatory covariates were removed from the final models. Julian date was the only covariate retained in the models for all antioxidant enzyme activities in the pectoralis and liver, but not for gene expression models. To test the hypothesis that dietary fat, dietary antioxidants, and flight training had an interactive effect on gene expression, we compared our global models to models including a three-way interaction between dietary fat, antioxidants, and training treatment. These models also test the two-way interactions between covariates. The models with the three-way interactions were not among the best fit models (i.e., within 3 ΔAIC scores of the global model), or in one case when they were (SOD enzyme activity), was not the most parsimonious model (4 fewer degrees of freedom); thus, we report results for only the main effects. See Supplementary data for full results from the linear models (Table S2, S3, S4).

#### Piecewise structural equation modeling

To test the hypothesis that NRF2 and PPAR transcription factors regulate antioxidant gene expression, and thereby regulate antioxidant enzyme activities we conducted a unidirectional path analysis that tests the causal relationships between regulatory genes (NRF2 and PPARs) and downstream antioxidant genes (CAT, SOD1, SOD2, GPX1, GPX4), and between antioxidant genes and antioxidant enzyme activities (CAT, SOD, GPx). We conducted a path analysis in the liver and pectoralis separately for flight-trained and untrained birds. We did not control for diet in our liver models since we found no effects of dietary fat and antioxidants (see the “Results” section). However, due to the positive effect of anthocyanins on select antioxidant gene expression in the pectoralis (see the “Results” section), we initially conducted path analyses for each anthocyanin diet in the pectoralis for flight-trained and untrained birds (Fig. 1). We present only the results from flight-trained and untrained birds (Fig. 6) since the causal relationships did not vary among antioxidant groups (Fig. S1). We used piecewise structural equation modeling (PSEM) using the PSEM R package (Lefcheck 2019) to calculate linear regression coefficients for each specified causal relationship in the causal model (Equations 1 and 2). Since we were not comparing causal models, we did not calculate the goodness-of-fit using tests of directed separation (“dsep,” Shipley 2016; Lefcheck 2019).

Causal Equation 1.

(1) \begin{eqnarray*} {Y_i} &=& {\beta _0} + {\beta _1}NRF2 + {\beta _2}PPAR\alpha + {\beta _3}PPAR\delta \,or\,PPAR\gamma \\ {Y_I} &=& {\rm{Genes:}}\,{\rm{CAT,}}\,{\rm{SOD1,}}\,{\rm{SOD2,}}\,{\rm{GPX1,}}\,{\rm{GPX4}} \end{eqnarray*}

Causal Equation 2.

(2) \begin{eqnarray*} {Y_I} &=& {\beta _0} + {\beta _1}CAT + {\beta _2}SOD1 + {\beta _3}SOD2\\ && +\, {\beta _4}GPX1 + {\beta _5}GPX4\\ {Y_i} &=& {\rm{Enzymes{:}}}\,{\rm{CAT,}}\,{\rm{SOD,GPx}}\end{eqnarray*}

## Results

### Flight training influences gene expression and enzyme activities in the liver and pectoralis (H1)

**Fig. 2:**
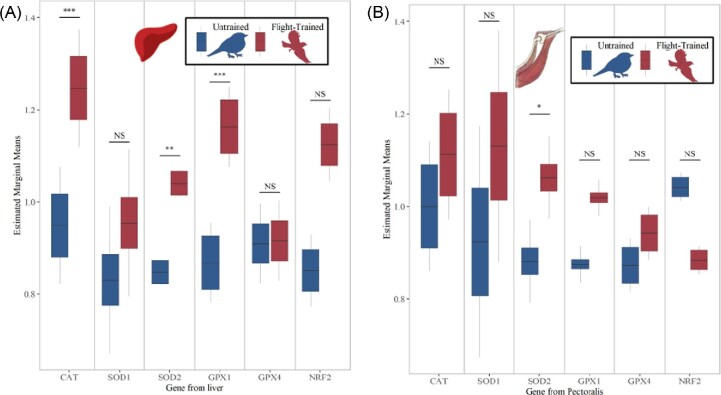
Relative gene expression (estimated marginal means from the linear mixed models; Table 1 and 2) in the **(A)** liver and **(B)** pectoralis muscle of European starlings that were (*N* = 49) or were not (*N* = 40) flown in the wind tunnel for 15 days. Antioxidant genes CAT, SOD1, and GPX1 in the liver, and SOD2 in the pectoralis, were expressed to the greatest extent in flight-trained birds compared with untrained birds. The asterisks correspond to significance levels *P < 0.1, **P < 0.05, ***P < 0.01 when comparing flight-trained and untrained birds.

Flight training consistently increased the expression of three of the five measured antioxidant enzyme genes in the liver (Fig. 2A) but affected only SOD2 in the pectoralis (Fig. 2B). In the liver, antioxidant gene expression of flight-trained starlings relative to untrained birds was greatest for CAT, SOD2, and GPX1 (Fig. 2A; CAT, T_87_ = 2.909, *P* = 0.0047; SOD2, T_87_ = 2.472, *P* = 0.016; GPX1, T_87_ = 2.904, *P* = 0.0047). Flight training did not significantly affect expression of SOD1 or GPX4 in the liver (Fig. 2A; SOD1, T_87_ = 0.912, *P* = 0.364; GPX4, T_87_ = 0.057, *P* = 0.955). The expression of transcription factors NRF2 (Fig. 2A) and PPARγ (Table 1) was greatest in liver of flight-trained birds, but this trend was not significant (NRF2, T_86_ = 1.327, *P* = 0.188; PPARγ, T_88_ = 1.432, *P* = 0.156) despite their effect sizes that were similar to the expression of CAT, SOD2, and GPX1 in response to flight training. In general, we found greater variability in the expression of transcription factors compared with antioxidant genes and this may have affected the detection of statistical significance. PPARα was not affected by flight training (Table 1; T_87_ = 1.517, *P* = 0.133). In the pectoralis, flight training increased the expression of only SOD2 (Fig. 2B; SOD2, T_89_ = 1.833, *P* = 0.070), decreased the expression of PPARδ (Table 2; T_88_ = −2.268, *P* = 0.026), and did not influence expression of the other four antioxidant genes (Fig. 2B; CAT, T_89_ = 1.101, *P* = 0.274; SOD1, T_88_ = 1.433, *P* = 0.156; GPX1, T_89_ = 1.180, *P* = 0.241) or NRF2 (Fig. 2B; T_89_ = −1.634, *P* = 0.106).

**Table 1:** Linear model results for antioxidant gene expression in the liver in relation to flight training, dietary antioxidant, and dietary PUFA. The intercept is the 13% 18:2n-6, Antioxidant Unsupplemented, Untrained Group (13U, UT). Data are reported as estimates (standard error) for each gene and the asterisks correspond to significance levels: *P < 0.1, **P < 0.05, ***P < 0.01.

	Dependent variable							
Covariate	CAT	SOD1	SOD2	GPX1	GPX4	NRF2	PPARγ^a^	PPARα^a^
Training (flight-trained)	0.298***	0.124	0.193**	0.295***	0.006	0.274	0.219	0.055
	(0.103)	(0.136)	(0.078)	(0.102)	(0.110)	(0.206)	(0.153)	(0.036)
Antioxidant (supplemented)	−0.176*	0.138	−0.053	−0.136	−0.117	−0.044	0.153	−0.041
	(0.102)	(0.135)	(0.077)	(0.101)	(0.110)	(0.204)	(0.152)	(0.036)
PUFA (32% 18:2n-6)	0.078	−0.181	−0.003	−0.037	0.058	0.113	−0.108	0.085**
	(0.102)	(0.135)	(0.078)	(0.101)	(0.110)	(0.204)	(0.152)	(0.036)
Intercept (13U, UT)	0.998***	0.852***	0.876***	0.955***	0.939***	0.816***	1.099***	1.036***
	(0.103)	(0.137)	(0.099)	(0.102)	(0.111)	(0.207)	(0.154)	(0.037)
Observations	87	87	87	87	87	86	87	87
*R* ^2^	0.124	0.044	0.072	0.109	0.017	0.025	0.043	0.098
Adjusted *R*^2^	0.092	0.009	0.039	0.076	−0.019	−0.011	0.008	0.066
Residual std. error	0.474 (df = 87)	0.628 (df = 83)	0.361 (df = 83)	0.469 (df = 83)	0.511 (df = 83)	0.946 (df = 82)	0.708 (df = 83)	0.168 (df = 83)
F Statistic	3.907** (df = 3; 83)	1.263 (df = 3; 83)	2.157* (df = 3; 83)	3.373** (df = 3; 83)	0.474 (df = 3; 83)	0.689 (df = 3; 82)	1.235 (df = 3; 83)	3.022** (df = 3; 83)

a Gene expressions for PPARδ and PPARα are presented in DeMoranville et al. (2020).

**Table 2:** Linear model results for antioxidant gene expression in the pectoralis in relation to flight training, dietary antioxidant, and dietary PUFA. The intercept is the 13% 18:2n-6, Antioxidant Unsupplemented, Untrained Group (13U, UT). Data are reported as estimates (standard error) for each gene and the asterisks correspond to significance levels: *P < 0.1, **P < 0.05, ***P < 0.01.

	Dependent variable							
Covariate	CAT	SOD1	SOD2	GPX1	GPX4	NRF2	PPARδ^a^	PPARα^a^
Training (flight-trained)	0.112	0.207	0.180*	0.226	0.069	−0.158	−0.296**	−0.019
	(0.104)	(0.144)	(0.098)	(0.141)	(0.095)	(0.097)	(0.130)	(0.051)
Antioxidant (supplemented)	0.215**	0.323**	0.079	0.173	0.024	0.049	−0.014	−0.021
	(0.101)	(0.144)	(0.098)	(0.140)	(0.095)	(0.096)	(0.130)	(0.051)
PUFA (32% 18:2n-6)	−0.067	−0.177	−0.099	−0.153	−0.091	0.012	0.047	0.022
	(0.101)	(0.144)	(0.098)	(0.140)	(0.095)	(0.096)	(0.130)	(0.051)
Intercept (13U, UT)	0.926***	0.851***	0.892***	0.880***	0.906***	1.011***	0.900***	1.008***
	(0.104)	(0.147)	(0.100)	(0.143)	(0.097)	(0.099)	(0.132)	(0.052)
Observations	89	88	89	89	89	89	88	89
*R* ^2^	0.068	0.095	0.057	0.019	0.018	0.067	0.060	0.006
Adjusted *R*^2^	0.035	0.063	0.024	0.016	−0.017	0.034	0.026	−0.029
Residual std. error	0.478 (df = 85)	0.674 (df = 84)	0.461 (df = 85)	0.572 (df = 85)	0.447 (df = 85)	0.420 (df = 85)	0.609 (df = 84)	0.239 (df = 85)
F Statistic	2.067 (df = 3; 85)	2.94** (df = 3; 84)	1.72 (df = 3; 85)	0.546 (df = 3; 85)	0.515 (df = 3; 85)	2.030 (df = 3; 85)	1.773 (df = 3; 84)	0.168 (df = 3; 85)

a Gene expressions for PPARγ and PPARα are presented in companion study DeMoranville et al. (2020).

**Fig. 3:**
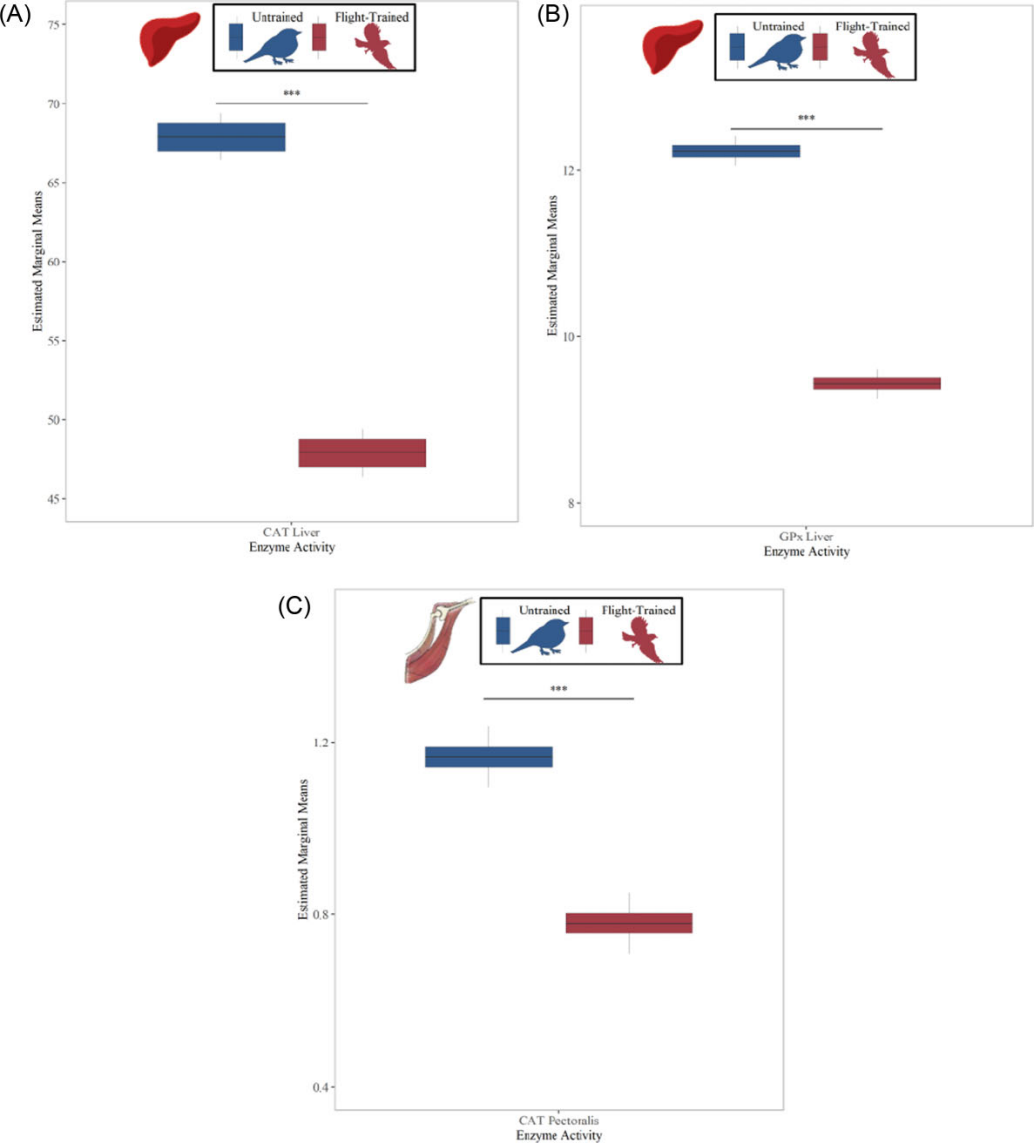
Antioxidant enzyme activities (estimated marginal means from the linear mixed models; Table 3) that were significantly influenced by flight training in the liver and pectoralis. CAT activity in the liver **(A)**, GPx activity in the liver **(B)**, and CAT activity in the pectoralis **(C)** were lowest in European starlings that were flown (*N* = 49) in the wind tunnel for 15 days compared with unflown birds (*N* = 40). There was no main effect of flight training on GPx or SOD activities in the pectoralis or on SOD activity in the liver (Table 2). The asterisks correspond to significance levels *P < 0.1, **P < 0.05, ***P < 0.01 between flight-trained and untrained birds.

Contrary to hypothesis 1, expression patterns of the antioxidant genes and antioxidant enzyme activities were not well coordinated in response to flight training in either tissue. In fact, antioxidant enzymes displayed an opposite pattern compared with antioxidant genes in relation to flight training. For example, GPx activity in the liver and CAT activity in the liver and pectoralis were lowest in flight-trained birds relative to untrained birds (Fig. 3A–C; GPx activity, T_86_ = −2.744, *P* = 0.0075; liver CAT activity, T_86_ = −4.093, *P* < 0.001; pectoralis CAT activity, T_90_ = −4.189, *P* < 0.001), while SOD activity in the liver and pectoralis and GPX activity in the pectoralis were unchanged by flight training (Table 3; liver SOD activity, T_86_ = −0.323, *P* = 0.747; pectoralis SOD activity, T_90_ = 1.779, *P* = 0.079; pectoralis GPx activity, T_90_ = −0.133, *P* = 0.894).

**Table 3:** Linear model results for change in antioxidant enzyme activities in the liver and pectoralis in relation to flight training, dietary antioxidant, and dietary PUFA. The intercept is the 13% 18:2n-6, Antioxidant Unsupplemented, Untrained Group (13U, UT). Data are reported as estimates (standard error) for each enzyme and the asterisks correspond to significance levels: *P < 0.1, **P < 0.05, ***P < 0.01.

	Dependent variable					
	Liver			Pectoralis		
Covariate	CAT nmol/min/mg	SOD U/mg	GPx nmol/min/mg	CAT nmol/min/mg	SOD U/mg	GPx nmol/min/mg
Training (flight-trained)	−20.029***	−0.033	−2.799***	−0.356***	0.342*	−0.038
	(4.894)	(0.102)	(1.020)	(0.092)	(0.192)	(0.282)
Antioxidant (supplemented)	−2.173	0.026	−0.210	0.077	0.178	−0.005
	(4.908)	(0.102)	(1.023)	(0.092)	(0.192)	(0.282)
						
PUFA (32% 18:2n-6)	0.790	−0.027	−0.145	−0.064	0.214	−0.132
	(4.869)	(0.101)	(1.015)	(0.092)	(0.191)	(0.281)
Julian date	−0.252*	−0.007**	−0.022	−0.007**	−0.018**	0.024***
	(0.141)	(0.003)	(0.029)	(0.003)	(0.005)	(0.008)
						
Intercept (13U, UT)	148.98***	3.884***	19.50**	3.335***	12.282***	−0.201
	(44.728)	(0.932)	(9.324)	(0.839)	(1.744)	(2.567)
Observations	86	86	86	90	90	90
*R* ^2^	0.199	0.067	0.091	0.231	0.1434	0.095
Adjusted *R*^2^	0.160	0.022	0.047	0.195	0.1036	0.053
Residual std. error	22.62 (df = 82)	0.4711 (df = 82)	4.715 (df = 82)	0.436 (df = 86)	0.9094 (df = 86)	1.336 (df = 86)
F Statistic	5.106*** (df = 4; 82)	1.473 (df = 4; 82)	2.058* (df = 4; 82)	6.439*** (df = 4; 86)	3.599*** (df = 4; 86)	2.254** (df = 4; 86)

### Julian date affects antioxidant enzyme activities but not antioxidant gene expression

Overall time on the experimental diets or the progression of the fall migratory season influenced antioxidant enzyme activities, but not antioxidant gene expression or the expression of transcription factors. When including Julian date as a continuous variable in our models, we observed a negative effect of Julian date on CAT and SOD activity in the liver and pectoralis (Table 3; liver CAT activity T_86_ = −1.790, *P* = 0.077; liver SOD activity T_86_ = −2.373, *P* = 0.020; pectoralis CAT activity T_90_ = −2.579, *P* = 0.012; pectoralis SOD activity T_90_ = −3.232, *P* = 0.0017), and a positive effect of date on GPx activity in the pectoralis (Table 3; T_90_ = 2.973, *P* = 0.004). There was no effect of date on GPx activity in the liver (Table 3; T_86_ = −0.022, *P* = 0.451). Our experimental design allowed us to determine the specific time intervals that affected antioxidant enzyme activity by utilizing cohorts that were sampled across the 3-month experiment. We constructed linear models using the experimental cohort (1–5) as a covariate instead of Julian date. In accordance with the effect of Julian date, the declines in CAT and SOD activity in the liver and pectoralis were primarily due to the last cohort sampled (Table 4), whereas the increase over the fall in GPx in the pectoralis was evident in the last two cohorts.

**Table 4:** The main effect of experimental cohort on antioxidant enzyme activities in the liver and pectoralis. The effects of dietary PUFA, dietary anthocyanin, and flight training from the linear models are not reported. The reported effects are relative to birds in Cohort 1 (in the 13% 18:2n-6, Antioxidant Unsupplemented, Untrained Group; 13U, UT). Asterisks correspond to significance levels: *P < 0.1, **P < 0.05, ***P < 0.01. Date ranges of final sampling for each cohort are reported in the footnote.^a^

	Δ Enzyme activity ± standard error (*P*-value) by Cohort in the Liver and Pectoralis							
	Liver				Pectoralis			
Cohort number	**2**	**3**	**4**	**5**	**2**	**3**	**4**	**5**
Enzyme activity:								
CAT	−16.46 ± 7.79 (0.038**)	−6.78 ± 7.29 (0.355)	−8.67 ± 7.51 (0.252)	−17.86 ± −2.38 (0.020**)	−0.075 ± 0.148 (0.615)	−0.257 ± −1.79 (0.078*)	−0.272 ± 0.062 (0.062*)	−0.307 ± 0.148 (0.041**)
SOD	−0.054 ± 0.166 (0.746)	−0.121 ± 0.155 (0.440)	−0.257 ± 0.160 (0.113)	−0.338 ± 0.160 (0.038**)	−0.377 ± 0.307 (0.223)	−0.207 ± 0.299 (0.490)	−0.544 ± 0.299 (0.072*)	−0.855 ± 0.308 (0.007***)
GPx	−2.73 ± 1.64 (0.100)	−1.32 ± 1.54 (0.394)	−1.31 ± 1.58 (0.410)	−1.93 ± 1.58 (0.227)	0.235 ± 0.450 (0.604)	0.633 ± 0.438 (0.152)	1.437 ± 0.438 (0.005***)	0.839 ± 0.451 (0.066*)

a Cohort final sampling occurred on the following dates: cohort 1, 17 October to 27 October; cohort 2, 29 October to 8 November; cohort 3, 10 November to 20 November; cohort 4, 22 November to 2 December; cohort 5, 4 December to 14 December.

### Differential effects of diet on gene expression: dietary anthocyanin increases pectoralis gene expression, yet no effect of dietary fat (H2, H3)

There were no main effects of dietary 18:2n-6 PUFA on gene expression of NRF2 or antioxidant gene expression in the liver (Table 1; NRF2, T_86_ = 0.554, *P* = 0.581; CAT, T_87_ = 0.768, *P* = 0.445; SOD1, T_87_ = −1.339, *P* = 0.184, SOD2, T_87_ = −0.037, *P* = 0.971; GPX1, T_87_ = −0.368, *P* = 0.714; GPX4, T_87_ = 0.526, *P* = 0.601) or in the pectoralis (Table 2; NRF2, T_89_ = 0.128, *P* = 0.899; CAT, T_89_ = −0.663, *P* = 0.509; SOD1, T_89_ = −1.232, *P* = 0.221, SOD2, T_89_ = −1.011, *P* = 0.315; GPX1, T_89_ = −0.334, *P* = 0.739; GPX4, T_89_ = −0.958, *P* = 0.341). PPARα was positively affected by dietary 18:2n-6 (Table 1; T_87_ = 2.363, *P* = 0.0205), and these results are discussed in a companion study (DeMoranville et al. 2020). In accordance with the lack of support for hypothesis 2, we found no support for an interactive effect of dietary 18:2n-6 and flight training on NRF2 or endogenous antioxidant genes or enzymes.

**Fig. 4:**
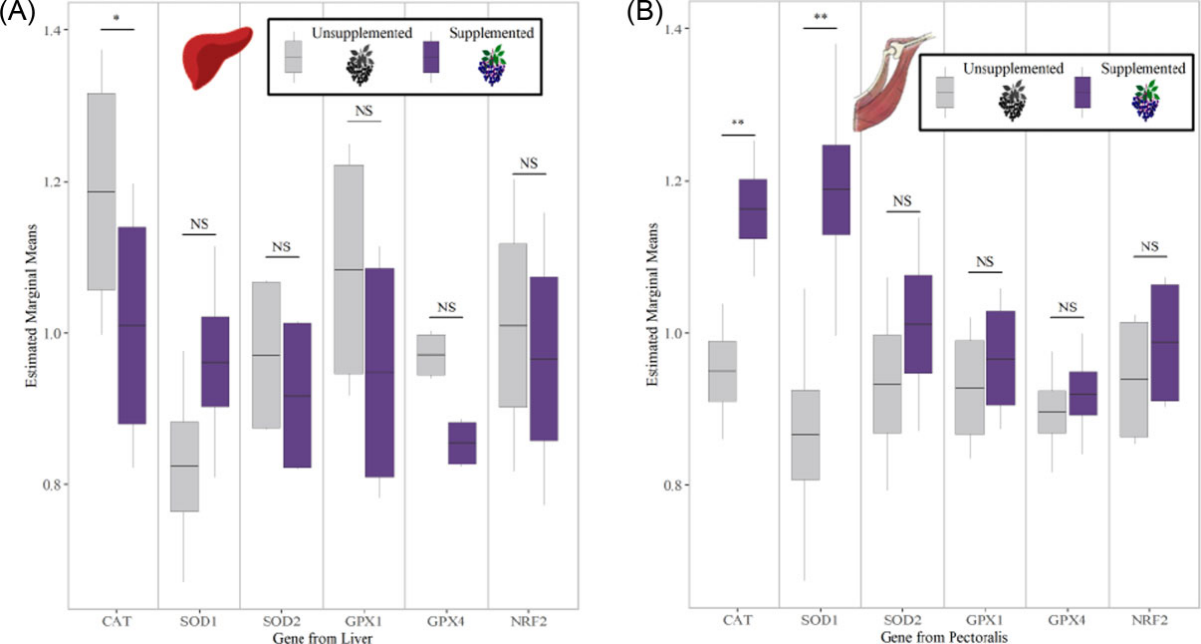
Relative gene expression (estimated marginal means from the linear mixed models; Table 1) in the **(A)** liver or **(B)** pectoralis muscle of European starlings that were (*N* = 45) or were not (*N* = 44) supplemented with the antioxidant, anthocyanin. **(B)** In the liver, antioxidant genes were not influenced by dietary anthocyanin. **(B)** In the pectoralis, antioxidant genes CAT and SOD1 were expressed to the greatest extent in anthocyanin-supplemented birds compared with unsupplemented birds. SOD2, GPX1, and GPX4 were not influenced by dietary anthocyanin. The asterisks correspond to significance levels *P < 0.1, **P < 0.05, ***P < 0.01 between anthocyanin-supplemented and unsupplemented birds.

Dietary anthocyanin had a more targeted effect on antioxidant genes compared with flight training. Dietary anthocyanin did not significantly influence NRF2 or antioxidant gene expression in the liver (NRF2, T_86_ = −0.262, *P* = 0.830; CAT, T_87_ = −1.734, *P* = 0.087; SOD1, T_87_ = 1.024, *P* = 0.309, SOD2, T_87_ = −0.690, *P* = 0.492; GPX1, T_87_ = −1.351, *P* = 0.180; GPX4, T_86_ = −1.063, *P* = 0.291) although there was a trend for CAT expression to be less in birds supplemented with anthocyanins (Fig. 4A). Consistent with the stimulatory hypothesis, anthocyanin-supplemented birds had greater CAT and SOD1 expression in the pectoralis relative to birds not supplemented with anthocyanins (Fig. 4B; CAT, T_89_ = 2.118, *P* = 0.0371; SOD1, T_88_ = 2.245, *P* = 0.0274), although there was no other effect of dietary anthocyanin on the other antioxidant genes or NRF2 expression (Fig. 4B; SOD2, T_89_ = 0.806, *P* = 0.423; GPX1, T_89_ = 0.320, *P* = 0.750; GPX4, T_89_ = 0.095, *P* = 0.799; NRF2, T_89_ = 0.507, *P* = 0.614).

### Path analysis—how the expression of NRF2 and PPAR transcription factors, antioxidant genes, and antioxidant enzyme activities interacts in flight-trained and untrained birds (H1)

**Fig. 5:**
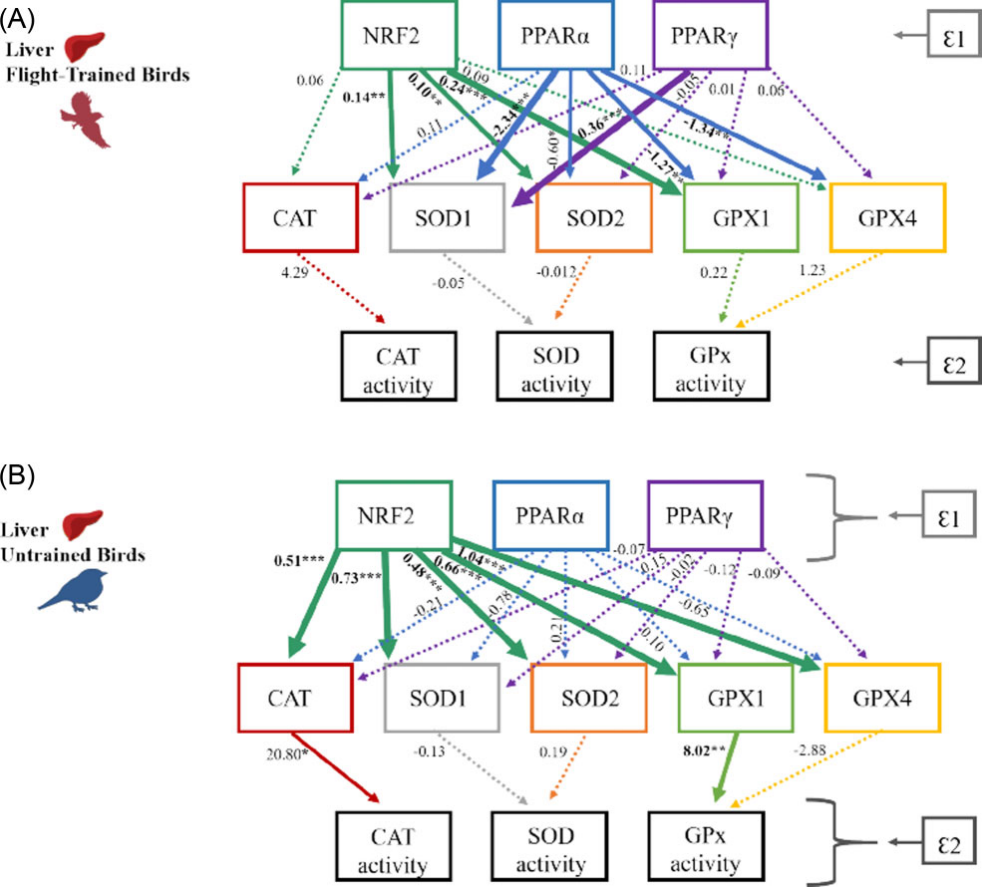
The casual structures to explain the hierarchical gene expression of transcription factors NRF2, PPARα, PPARγ on the expression of downstream antioxidant target genes (CAT, SOD1, SOD2, GPX1, GPX4) and antioxidant enzyme activity (CAT, SOD, GPx) in the liver of **(A)** flight-trained European starlings and **(B)** untrained starlings. Ɛ1 represents all unmeasured variables that could affect NRF2 and PPAR gene expression (e.g., ligand type and quantity, cofactors) while Ɛ2 represents all unmeasured variables that could affect enzyme activities (e.g., posttranslational modifications). Path estimates are reported to the left of each line for all causal relationships. The dashed lines indicate nonsignificant causal relationships (all *P*-values >0.1). Solid lines indicate significant causal relationships, and the asterisks and line thickness correspond to significance levels: *P < 0.1, **P < 0.05, ***P < 0.01. In general, flight training concentrated the regulation of NRF2 from all antioxidant genes (in untrained birds) to 60% of genes and initiates a new negative relationship between PPARα and 80% of the antioxidant genes. Antioxidant enzyme activities were not significantly related to antioxidant gene expression in flight-trained birds, although CAT and GPx enzyme activities were significantly related to antioxidant gene expression in untrained birds.

**Fig. 6:**
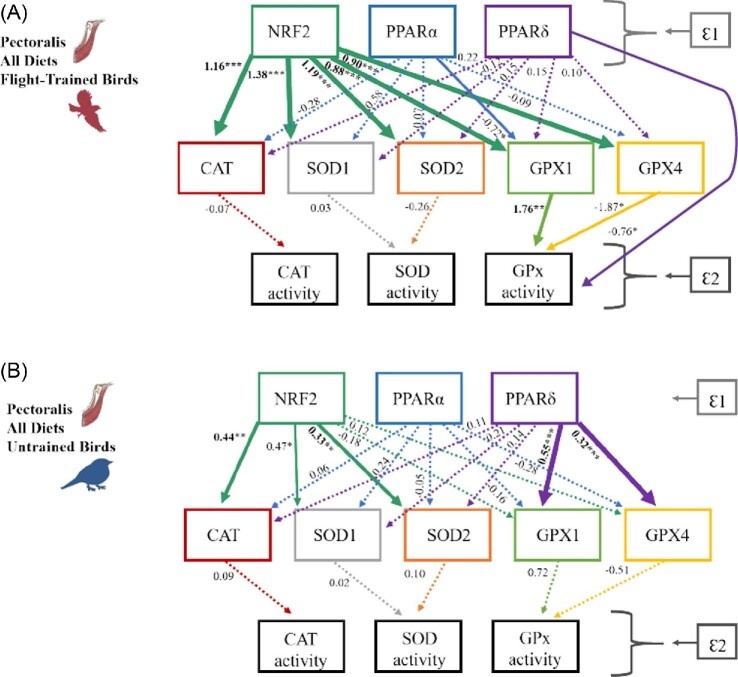
The casual structures to explain the hierarchical gene expression of transcription factors NRF2, PPARα, PPARδ on the gene expression of downstream antioxidant target genes (CAT, SOD1, SOD2, GPX1, GPX4) and antioxidant enzyme activity (CAT, SOD, GPx) in the pectoralis of **(A)** flight-trained European starlings and **(B)** untrained starlings consuming all diets. Ɛ1 represents all unmeasured variables that could affect NRF2 and PPAR gene expression (e.g., ligand type and quantity, cofactors), while Ɛ2 represents all unmeasured variables that could affect enzyme activities (e.g., posttranslational modifications). Path estimates are reported to the left of each line for all causal relationships. The dashed lines indicate nonsignificant causal relationships (all *P*-values >0.1). Solid lines indicate significant causal relationships, and the asterisks and line thickness correspond to significance levels: *P < 0.1, **P < 0.05, ***P < 0.01. Flight training strengthens the relationships between NRF2 and antioxidant genes and broadens them to include all antioxidant genes. Flight training alters PPAR regulation on select antioxidant genes; PPARα and PPARδ negatively influence GPX1 expression and GPx activity, respectively, in flight-trained birds, while PPARδ positively influences GPX expression in untrained birds.

In order to test hypothesis 1, that flight training increases (1) NRF2 and PPAR expression and thereby increases (2) the expression of their antioxidant genes and the (3) activities of their related antioxidant enzyme activities, we constructed a causal model for flight-trained and untrained birds for both the liver (Fig. 5) and pectoralis (Fig. 6). The regression coefficient associated with each causal relationship between the transcription factors and the antioxidant genes indicates the extent of change in the number of transcripts of the antioxidant genes (CAT, SOD1, SOD2, GPX1, GPX4) for each one-transcript change in NRF2 and PPAR transcription factors (NRF2, PPARα, PPARγ, PPARδ). For example, for the liver of flight-trained birds (Fig. 5A), one of the strongest causal relationships showed that a one-transcript change in PPARα resulted in a 2.34 transcript decrease in the SOD1 gene. Similarly, the regression coefficients between the antioxidant genes and antioxidant enzyme activities represent a 1-unit change in enzyme activity for each one-transcript change in antioxidant genes.

In general, flight training altered the relationships among transcription factors and antioxidant genes in a tissue-specific manner. For example, in the liver (Fig. 5), flight training concentrated the regulation of NRF2 from all antioxidant genes in untrained birds to 60% of genes in flight-trained birds. In addition, flight training initiated a new positive relationship between PPARγ and SOD1, and new negative relationships between PPARα and 80% of the antioxidant genes (Fig. 5). In contrast, in the pectoralis (Fig. 6), flight training strengthened the relationships between NRF2 and all antioxidant genes compared with only three significant relationships in untrained birds. In addition, flight training altered PPAR regulation of select antioxidant genes. Specifically, PPARα and PPARδ negatively influenced GPX1 expression and GPx activity, respectively in flight-trained birds while PPARδ positively influenced GPX1 and GPX4 expression in untrained birds. These relationships were maintained in birds consuming different amounts of dietary antioxidants (Fig. S1) despite the significant positive effect of dietary anthocyanin on select genes in the pectoralis; thus, diet groups are combined for flight-trained and untrained birds in Fig. 6.

We found little evidence that antioxidant gene expression related to antioxidant enzyme activities. For example, in the liver (Fig. 5), antioxidant enzyme activities were not significantly related to antioxidant gene expression in flight-trained birds, although CAT and GPx enzyme activities were positively related to CAT and GPX1 expression, respectively, in untrained birds. In the pectoralis (Fig. 6), GPx activity was positively influenced by GPX1 activity in flight-trained birds, although we detected no other significant relationships among the measured antioxidant genes and enzymes in flight-trained or untrained birds suggesting posttranslational modifications to these antioxidant proteins.

## Discussion

### Tissue-specific differences in gene expression and enzyme activity patterns in response to flight training (H1)

We found support for hypothesis 1 (flight training stimulated the expression of antioxidant genes) that was tissue specific: flight-trained birds had significantly higher expression of three of the five antioxidant genes (CAT, SOD2, GPX1) measured in liver but only one of five (SOD2) measured in pectoralis. The mitochondria produce superoxide radicals that are among the most reactive (Halliwell and Gutteridge 2007), and the need to convert superoxide to hydrogen peroxide is potentially why SOD2 expression was highest in both the liver and pectoralis. Furthermore, SOD2 is localized at the mitochondrial site of reactive species production, whereas SOD1 is localized in the cytosol and mitochondrial intermembrane space (Halliwell and Gutteridge 2007; Powers and Jackson 2008; Cooper-Mullin and McWilliams 2016), which may explain the lack of upregulation of SOD1 in response to flight training. GPX and CAT are the next line of antioxidant defense after SOD2 in that they reduce the newly converted hydrogen peroxide radical. GPX1 is also localized in the mitochondria and its expression was highest in the liver of flight-trained birds, whereas there was no difference in GPX4 expression. We predicted that GPX4 would be crucial to flying birds that rely on fat as fuel since GPX4 is localized in the nuclei and mitochondria and is the only isoform that can act on peroxidized fatty acid residues (Halliwell and Gutteridge 2007; Powers and Jackson 2008; Cooper-Mullin and McWilliams 2016); thus, we are unsure why birds do not also upregulate GPX4. CAT is exclusively located in peroxisomes, a crucial site of lipid oxidation (Lodhi and Semenkovich 2014). Perhaps upregulation of CAT in the liver of flight-trained birds reduced the negative effects of lipid oxidation within peroxisomes, although why this did not also occur in pectoralis muscle is puzzling. The transcription factors that we measured (NRF2, PPARα, PPARγ) were not differentially expressed in either tissue in response to flight training with one exception: PPARδ expression was lower in the pectoralis of flight-trained birds possibly reflecting its regulatory roles in lipid storage rather than lipid mobilization (Bindesbøll et al. 2013; Chayama et al. 2019; DeMoranville et al. 2020). Future studies should investigate whether flight training increases the transcriptional activity of NRF2 or PPARs.

Our causal models (Figs. 5 and 6) provided some evidence for the regulatory pathway proposed in hypothesis 1, that flight training increases (1) NRF2 and PPAR expression and thereby increases (2) the expression of their antioxidant genes and the (3) activities of their related antioxidant enzyme activities. Our causal models indicated that NRF2 mediates the expression of a minimum of 60% of the antioxidant genes measured here. Importantly, flight training altered the relationships among NRF2, PPARs and antioxidant genes in a tissue-specific manner. In the liver, flight training concentrated the regulation of NRF2 from all antioxidant genes in the untrained state to 60% of genes (SOD1, SOD2, GPX1) in the flight-trained state. In contrast, in the pectoralis, flight training broadened the relationships between NRF2 and the antioxidant genes from 60% of the antioxidant genes (CAT, SOD1, SOD2) in untrained birds to all five measured antioxidant genes when flight trained. Similarly, the NRF2 pathway was significantly upregulated in Burmese pythons (*Python molurus bivittatus*) post-feeding during which rapid organ growth and 44-fold increases in metabolism occur (Andrew et al. 2017). In contrast to our study, NRF2 and all three antioxidant enzymes (CAT, SOD, and GPX1) were consistently upregulated in the small intestine, kidney, and liver of Burmese pythons indicating that NRF2 activated the three enzymes likely to protect all tissues from increases in reactive species production associated with increases in metabolism (Andrew et al. 2017). These contrasting results could be due to differences between studies in the focal tissues or species, the cause of metabolic increases (exercise vs. organ growth), or the scale of the measurements (i.e., our RT-qPCR analyses, transcriptome analysis used for the python study).

Some support for the proposed regulatory pathway (H1) was also provided by the PPAR transcription factors, but again the patterns were tissue specific (Figs. 5 and 6). In the liver, flight training initiated a new positive relationship between PPARγ and SOD1 and new negative relationships between PPARα and 80% of the antioxidant genes. In the pectoralis, PPARα and PPARδ negatively influenced GPX1 expression and GPx activity in flight-trained birds while PPARδ positively influenced GPX expression in untrained birds. In mammals, PPARs directly transcribe CAT, GPX, and SOD (Kim and Yang 2013), but it remains unknown how changes in their transcription relate to expression of downstream antioxidant target genes. We found that flight training clearly initiated PPAR regulatory involvement in songbirds; however, it is unclear how PPAR expression influences individual antioxidant enzyme expression. Transcriptome studies that characterize a complete set of differentially expressed genes will provide a more holistic picture of gene expression patterns and help to elucidate pathway responses to flight.

In contrast to our predictions, our causal models (Figs. 5 and 6) provided little evidence that flight training elicited a corresponding increase in both the expression of antioxidant genes and the activities of their related antioxidant enzyme activities. In general, the gene expression of antioxidant enzymes did not consistently positively correlate with enzymatic activities. Recent advances in sequencing and proteomics reveal that mRNA expression and protein abundance diverge during times of osmolarity stress and oxidative stress (Vogel and Marcotte 2012); thus, exercise may disrupt the correlative relationship between the two measures. Further exploration of protein and enzyme regulation in perturbed systems is warranted (Vogel and Marcotte 2012), and we offer two additional plausible scenarios to investigate. This lack of correspondence between gene expression and activity of antioxidant enzymes may occur because our measures of enzyme activities combine that for all isoforms that may mask the direct regulatory relationships among specific gene isoforms. Furthermore, enzyme activities measure the enzyme concentration at a given time and provide no information on enzymatic flux within a metabolic network (Fell 2005). The metabolic flux, or turnover, of the antioxidant enzymes may more closely reflect corresponding gene expression levels that may also be influenced by circadian rhythms (Lück et al. 2014). The exceptions to this general lack of association between gene expression and activity of antioxidant enzymes included two positive relationships in the liver of untrained birds (Fig. 5B: CAT, GPX1) and two negative relationships in the pectoralis of flight-trained birds (Fig. 6A: GPX1, GPX4). We speculate that CAT and GPX expression may be more tightly linked to enzymatic activities due to their importance in reducing the more common hydrogen peroxide radical compared with SOD that neutralizes superoxide that is rapidly converted to hydrogen peroxide. The need to maintain tight regulation of CAT and GPx is also suggested by the lower antioxidant enzyme activities in flight-trained birds compared with untrained birds in the liver (CAT, GPx) and pectoralis (CAT) (Fig. 3) that may have occurred via posttranslational modifications to proteins to inactivate CAT and GPx once birds had recovered from flight training (2 days after the last flight). Birds can rapidly adjust antioxidant enzymes as shown by increases during a single flight (Cooper-Mullin et al. 2019; Dick and Guglielmo 2019; Frawley et al. 2021a; McWilliams et al. 2021), and given this short response time it is unsurprising that enzyme activities in this study are lower in flight-trained birds 2 days after flight compared with unflown birds. Consistent with these results, Frawley et al. 2021b employed a similar experimental design and observed lower GPx activity in the heart and SOD activity in the liver in trained starlings compared with starlings not trained in a wind tunnel during recovery. Research on how antioxidant capacity recovers after flight is emerging, and remains to be mechanistically explained (McWilliams et al. 2021).

### Dietary fat quality does not affect molecular antioxidant pathways (H2)

Our study does not provide evidence to support H2 that migratory songbirds fed diets composed of more 18:2n-6 PUFA are more susceptible to oxidative damage and thus have increased expression levels of NRF2 and PPAR transcription factors, select antioxidant genes, and corresponding antioxidant enzyme activities compared with when fed diets with less 18:2n-6. This hypothesis was informed by the biochemistry and oxidative susceptibility of PUFA (Skrip and McWilliams 2016) and the ability of 18:2n-6 to stimulate antioxidant enzymes in fish (Li et al. 2013; Zengin and Yilmaz 2016). Rainbow trout (*Oncorhynchus mykiss*) larvae with the highest PUFA fatty acid compositions in the first week of growth had the highest levels of CAT, SOD, and GPX expression compared with later growth periods with lower PUFA composition (Zengin and Yilmaz 2016), and Darkbarbel catfish (*Pelteobagrus vachelli*) fed higher levels of linseed oil containing more PUFA had higher serum antioxidant enzyme activities (CAT, SOD, GPX) (Li et al. 2013). It is possible that our dietary 18:2n-6 composition did not oxidatively challenge birds even after flight training, and thereby there was no need for birds to upregulate antioxidant genes or enzymes. Consistent with our results, gene expression levels of CAT, SOD1, SOD2, and GPX were similar among rats fed different sources of n-3 and n-6 PUFA or fed no PUFA (Tou et al. 2011). Lipid peroxidation remains a particularly relevant challenge for migratory songbirds that rely on 18:2n-6 and other fats to fuel migratory flights (Pierce et al. 2004; Pierce and McWilliams 2005; Price et al. 2008; Smith and McWilliams 2010). Future studies that compare the effect of different dietary 18:2n-6 levels on endogenous antioxidants and that simultaneously measure oxidative damage will be able to better elucidate the effects of dietary 18:2n-6 on the endogenous antioxidant system in migratory birds.

### Dietary antioxidants stimulate antioxidant gene expression (H3) in the pectoralis but not liver

In accordance with hypothesis 3, we found evidence for a stimulatory effect of dietary anthocyanins on antioxidant pathways, but contrary to predictions this occurred only in the pectoralis muscle independent of flight training. We expected that when birds were metabolically challenged by flight training, dietary anthocyanins would quench excess reactive species and disrupt NRF2 and PPAR signaling to have an inhibitory or compensatory effect of dietary anthocyanins on enzyme activities (Gomez-Cabrera et al. 2008; Ristow et al. 2009; Done and Traustadóttir 2016). Instead, we observed that dietary anthocyanins maintained their stimulatory properties, likely through NRF2 (Shih et al. 2007; Cimino et al. 2013; Aboonabi and Singh 2015; Tian et al. 2019; Aboonabi et al. 2020), to increase the gene expression of antioxidant enzymes (CAT, SOD1) in the pectoralis of flight-trained and untrained birds. Given this stimulatory effect was limited to the flight muscle and not the liver, dietary antioxidants such as anthocyanins may be crucial signaling molecules especially in metabolically active tissues directly involved in responding to some challenge (e.g., flight training). Like exercise, an immune challenge produces reactive species at the site of injury that act as signaling molecules to recruit inflammatory mediators (e.g., cytokines, prostaglandins) and endogenous antioxidants for repair (Ahmed et al. 2017). Dietary anthocyanins increased the likelihood of mounting an immune response after an inflicted immune-challenge in Blackcaps (*Sylvia atricapilla*) (Catoni et al. 2008) perhaps through the amelioration of reactive species either directly or by stimulating endogenous antioxidants. Considered together, birds consuming dietary anthocyanins appear to gain protective benefits in response to immunological and exercise-related challenges, both of which are directly relevant to birds during migration. Furthermore, dietary anthocyanins reduced the production of corticosterone (CORT) in flying songbirds (Casagrande et al. 2020) indicating that antioxidant consumption protects against the metabolic costs associated with high glucocorticoid levels, like reactive species production. The exact mechanisms responsible for the observed antioxidant-protective effects in birds have yet to be elucidated. Future studies should investigate the protective effects of anthocyanin supplementation in migratory songbirds by characterizing the interactions between the NRF2 antioxidant pathway, NF-κB immune pathway (Ahmed et al. 2017), and the hypothalamic–pituitary–adrenal axis responsible for glucocorticoid production.

### Possible mechanisms to explain tissue-specific differences in antioxidant gene expression

We propose that the distinct gene expression profiles for the antioxidant genes for the two tissues are driven by the metabolic state and main functions of the liver and pectoralis. A companion study demonstrated that key genes involved in fat metabolism were upregulated in the pectoralis of flight-trained songbirds, but not in the liver (DeMoranville et al. 2020). In contrast, we demonstrated here that flight training upregulated antioxidant genes in the liver but not in the pectoralis. The pectoralis may require an upregulation of metabolic genes to match the higher demands of flight training (Mcfarlan et al. 2009; Zhang et al. 2015; Corder et al. 2016; DeMoranville et al. 2019) compared with the liver that also functions as an endocrine and exocrine gland. Additionally, the tissue-specific protein turnover rates of the liver are two times faster than the protein turnover rates of the pectoralis in migratory birds (Bauchinger and McWilliams 2010), and this may have allowed the liver to advance from a “metabolic state” that prioritizes the expression of genes involved in fat metabolism to a “repair and recovery state” that prioritizes the expression of antioxidant and pro-inflammatory genes (Wilson and Johnson 2000; Pillon Barcelos et al. 2017) within 2 days after the longest flight. In contrast, the relatively slower turnover of the pectoralis suggests that this muscle may have remained in a metabolic state that prioritizes the expression of genes involved in fat metabolism and had not yet transitioned to the expression of antioxidant genes. In general, the time course of antioxidant gene expression changes according to tissue type, kinetics within a tissue, amount of damage generated, and with exercise (reviewed in Costantini 2019). For example in ground squirrels, CAT, SOD1, SOD2, and GPX1 were differentially expressed among different skeletal muscle types (i.e., soleus, extensor digitorum longus, gastrocnemius) potentially reflecting metabolic differences in slow-twitch, fast-twitch, and mixed muscles (Wei et al. 2019). We propose that flight-trained birds consuming anthocyanins may have been able to upregulate antioxidant genes CAT and SOD1 in addition to metabolic genes (DeMoranville et al. 2020) because these birds incurred an energy saving, perhaps through the reduction of the glucocorticoid CORT (Casagrande et al. 2020). Dietary anthocyanins did not have a stimulatory effect on antioxidant gene expression in the liver perhaps because flight training was driving increases in these enzymatic genes. Similarly, there may be less spare capacity for antioxidant genes to be upregulated by dietary antioxidants because the antioxidant pathways are already operating at a high level. In sum, evidence to date from this study and our companion study (DeMoranville et al. 2020) suggests that flight training and dietary antioxidants, less so dietary fat, strongly affect the gene-level regulation of the antioxidant system and fat metabolism, that these effects are tissue-specific, and likely explained by functional differences between tissues as well as fundamental differences in their turnover rates.

## Relevance and significance

We provide some of the first evidence for how antioxidant pathways respond to ecological factors that are relevant to songbirds during migration. Exercise, dietary fat, and dietary antioxidants have been shown to influence antioxidant transcription factors (NRF2, PPARs) and their downstream antioxidant genes in other organisms (Ristow et al. 2009; Wang 2010; Done and Traustadóttir 2016), although ours is the first study that investigates all three simultaneously in a wild-caught migratory songbird. Our study confirms that repeated bouts of flight and dietary antioxidants, but not dietary fat, stimulate the downstream expression of select antioxidant genes and by inference the transcriptional activity of NRF2 and PPARs in starlings. Given that birds and their relatives have a constituently active NRF2 because of unique mutations in the KEAP1 repressor gene (Castiglione et al. 2020), ecological factors such as exercise and diet quality may be more crucial in modulating the transcriptional activity of NRF2, independent of any effect on KEAP1, and thus the expression of genes involved in antioxidant protection, as we have shown. Birds during migration are quite selective in what they eat (Pierce et al. 2004; Pierce and McWilliams 2005; Price et al. 2008; Schaefer et al. 2008; Smith and McWilliams 2010; Alan et al. 2013; Bolser et al. 2013), and these diet choices directly affect their supply of nutrients and energy but also, as we have shown, can affect the regulation of key antioxidant pathways. Likewise, birds during migration undergo regular, often daily flights interrupted by periods at stopover sites as they travel between breeding and wintering areas. Our results suggest that flying itself directly affects the regulation of key metabolic pathways involved in antioxidant protection, and a companion study has shown the same for pathways involved in fat metabolism (DeMoranville et al. 2020). It remains to be demonstrated how the extent of these ecological factors (i.e., intensity or duration of flight, amounts of dietary antioxidants) influences these effects on key metabolic pathways.

## Supplementary Material

obab035_Supplemental_File
