# Supplementary material for: Flight and Dietary Antioxidants Influence Antioxidant Expression and Activity in a Migratory Bird
Source: Integr Org Biol. 2021 Dec 30;4(1):obab035. doi: 10.1093/iob/obab035 (PMC8802218; doi:10.1093/iob/obab035)
Supplement: obab035_Supplemental_File [file obab035_supplemental_file.docx]

| **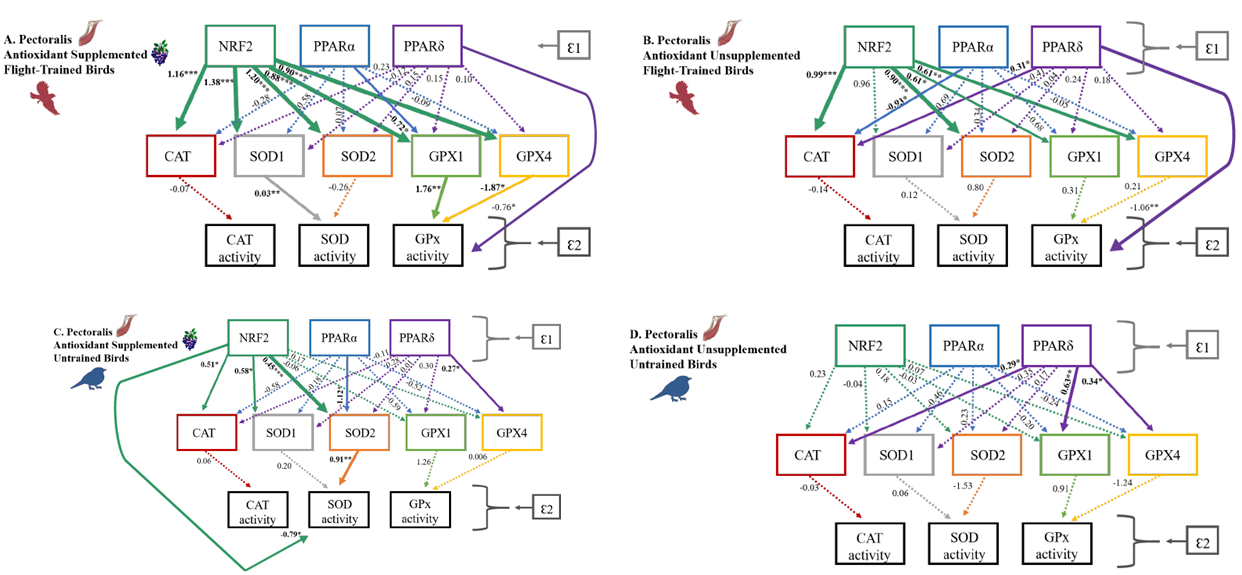**  **Figure S1.**  The casual structures to explain the hierarchical gene expression of transcription factors NRF2, PPARα, PPARδ on the gene expression of downstream antioxidant target genes (CAT, SOD1, SOD2, GPX1 and GPX4) and antioxidant enzyme activity (CAT, SOD, GPx) in the pectoralis of **A.)** anthocyanin supplemented flight-trained European starlings **B.)** anthocyanin unsupplemented flight-trained starlings **C.)** anthocyanin supplemented untrained starlings **D.)** anthocyanin unsupplemented untrained starlings.Ɛ1 represents all unmeasured variables that could affect NRF2 and PPAR gene expression (e.g. ligand type and quantity, cofactors) while Ɛ2 represents all unmeasured variables that could affect enzyme activities (e.g. post translational modifications). Path estimates are reported to the left of each line for all causal relationships. The dashed lines indicate non-significant causal relationships (all p-values>0.1). Solid lines indicate significant causal relationships, and the asterisks and line thickness correspond to significance levels: *p<0.1, ****p<0.05, ***p<0.01.** Dietary anthocyanin does not strongly alter the relationships among transcription factors, antioxidant genes, and antioxidant enzymes. Whereas, flight training strengthens the relationships between NRF2 and antioxidant genes and broadens them to include all antioxidant genes. Flight training alters PPAR regulation on select antioxidant genes: PPARα and PPARδ differentially influence CAT expression in flight-trained birds while PPARδ positively influences GPX expression in untrained birds. |  |
| --- | --- |

**Table S1**. Sequences of primers used for reverse transcription quantitative PCR in the liver and pectoralis muscle of European Starlings.

| Gene | Forward and Reverse Primers | | RefSeq accession number | |
| --- | --- | --- | --- | --- |
| β-actin | F: 5’- GCTACGAACTCCCTGATGG -3’ R: 5’- GACTCCATACCCAGGAAAGATG -3’ | XM_014870664.1 | |  |
| CAT | F: 5’- TCATTCAGAAACGAGCTGTGA - 3’ R: 5’- CCCACCCTCAGCATTGTATT - 3’ | XM_014878840.1 | |  |
| SOD1 | F: 5’- CCGGTGAAAGTCACTGGAAA - 3’ R: 5’- GTGCAGCCATTAGTGTTGTC - 3’ | XM_014888729.1 | |  |
| SOD2 | F: 5’- GCAAGGAACAACAGGTCTCA - 3’ R: 5’- TCACATTCCAGATGGCTTTCA - 3’ | XM_014874170.1 | |  |
| GPX1 | F: 5’- CCAGTTCGGTCACCAGGAAA -3’ R: 5’- CGCACTTCTCGAACAGGATG -3’ | XM_014869607.1 | |  |
| GPX4 | F: 5’- ACTTCACCAAGTTCCTCATTAACC -3’ R: 5’- CTCGATCACATAGGGATCTTCCA -3’ | XM_014882939.1 | |  |
| NRF2 | F: 5’- TGGAATTAGACGAAGAGACAGGTGA -3’ R: 5’- TTTGAAGGCTCTGTGGTCTGTGA -3’ | XM_014890038.1 | |  |
| PPARα | F: 5’- GTGCGTGACATCAAGGAGAA -3’ R: 5’- GGTGTCATCAGGATGGTTGT -3’ | XM_014880503.1 | |  |
| PPARδ | F: 5’- GCATGTCACACAACGCAAT -3’ R: 5’- GATCTCGCTTGCCGTCAG -3’ | XM_014872751.1 | |  |
| PPARγ | F: 5’- ACGACTCCTACATCAAATCCTT -3’ R: 5’- ACGAAGGGTGATTTGTCTGT -3’ | XM_014869773.1 | |  |

| **Table S2.** Mean gene expression of each antioxidant gene in the liver relative to the reference group^R^, 13U, UT used for the linear models reported in text and in Table 2. Relative mean gene expression ± standard deviation are reported for each treatment group, and treatment groups are abbreviated as follows: 13 or 32% PUFA, Unsupplemented (U) or Supplemented (S) with antioxidants, and Untrained (UT) or Flight-Trained (T) for training. The number of individuals for each treatment groups are reported in the first row. | | | | | | | | |  |
| --- | --- | --- | --- | --- | --- | --- | --- | --- | --- |
|  | *Relative Mean Gene Expression* ± *Standard deviation*  *by Diet and Flight Training Treatment Groups in the Liver:* | | | | | | | |  |
| *Liver Antioxidant Genes* | 13U, UT^R^ | | 13U, T | 13S, UT | 13S, T | 32U, UT | 32U, T | 32S, UT | 32S, T |
| *Number of Individuals* | *10* | | *13* | *10* | *13* | *10* | *11* | *8* | *12* |
| CAT | 1.00 ± 0.29 | | 1.26 ± 0.59 | 0.86 ± 0.34 | 1.13 ± 0.31 | 1.01 ± 0.57 | 1.47 ± 0.68 | 0.93 ± 0.49 | 1.14 ± 0.43 |
| SOD1 | | 1.00 ± 0.79 | 0.85 ± 0.72 | 0.73 ± 0.42 | 1.33 ± 0.71 | 0.75 ± 0.57 | 0.74 ± 0.50 | 0.85 ± 0.74 | 0.89 ± 0.45 |
| SOD2 | 1.00 ± 0.33 | | 1.02 ± 0.47 | 0.73 ± 0.22 | 1.04 ± 0.31 | 0.86 ± 0.41 | 1.02 ± 0.35 | 0.79 ± 0.31 | 1.08 ± 0.41 |
| GPX1 | 1.00 ± 0.36 | | 1.21 ± 0.47 | 0.74 ± 0.24 | 1.19 ± 0.46 | 0.92 ± 0.48 | 1.22 ± 0.77 | 0.83 ± 0.47 | 1.04 ± 0.39 |
| GPX4 | 1.00 ± 0.42 | | 0.91 ± 0.48 | 0.72 ± 0.34 | 0.90 ± 0.44 | 1.10 ± 0.73 | 0.89 ± 0.54 | 0.81 ± 0.46 | 0.95 ± 0.63 |
| NRF2 | 1.00 ± 0.55 | | 1.08 ± 0.62 | 0.71 ± 0.21 | 0.96 ± 0.95 | 0.94 ± 0.70 | 1.04 ± 0.60 | 0.72 ± 0.41 | 1.41 ± 2.00 |
| PPARγ | 1.00 ± 0.44 | | 1.44 ± 1.10 | 1.44 ± 0.80 | 1.29 ± 0.66 | 0.96 ± 0.43 | 1.19 ± 0.50 | 1.07 ± 0.68 | 1.46 ± 0.72 |
| PPARα | 1.00 ± 0.21 | | 1.10 ± 0.22 | 1.06 ± 0.08 | 1.02 ± 0.21 | 1.11 ± 0.16 | 1.21 ± 0.22 | 1.06 ± 0.06 | 1.13 ± 0.21 |

| **Table S3.** Mean gene expression of each antioxidant gene in the pectoralis muscle relative to the reference group^R^, 13U, UT used for the linear models reported in text and in Table 3. Relative mean gene expression ± standard deviation are reported for each treatment group, and treatment groups are abbreviated as follows: 13 or 32% PUFA, Unsupplemented (U) or Supplemented (S) with antioxidants, and Untrained (UT) or Flight-Trained (T) for training. The number of individuals for each treatment groups are reported in the first row or in the footnote^a^. | | | | | | | | |  |
| --- | --- | --- | --- | --- | --- | --- | --- | --- | --- |
|  | *Relative Mean Gene Expression* ± *Standard deviation*  *by Diet and Flight Training Treatment Groups in the Pectoralis:* | | | | | | | |  |
| *Pectoralis Antioxidant Genes* | 13U, UT^R^ | | 13U, T | 13S, UT | 13S, T | 32U, UT | 32U, T | 32S, UT | 32S, T |
| *Number of Individuals* | *10* | | *13* | *10* | *13* | *10* | *11* | 10 | *12* |
| CAT | 1.00 ± 0.25 | | 1.02 ± 0.49 | 1.10 ± 0.51 | 1.25 ± 0.53 | 0.79 ± 0.47 | 0.99 ± 0.42 | 1.11 ± 0.56 | 1.20 ± 0.56 |
| SOD1 | | 1.00 ± 0.57 | 1.03 ± 0.79^a^ | 0.94 ± 0.42 | 1.47 ± 0.77 | 0.62 ± 0.59 | 0.83 ± 0.56 | 1.14 ± 0.81 | 1.19 ± 0.76 |
| SOD2 | 1.00 ± 0.31 | | 1.07 ± 0.35 | 0.89 ± 0.49 | 1.14 ± 0.51 | 0.67 ± 0.33 | 0.99 ± 0.38 | 0.97 ± 0.46 | 1.06 ± 0.72 |
| GPX1 | 1.00 ± 0.35 | | 0.95 ± 0.34 | 0.73 ± 0.21 | 1.17 ± 0.41 | 0.86 ± 0.45 | 0.93 ± 0.34 | 0.91 ± 0.25 | 1.02 ± 0.32 |
| GPX4 | 1.00 ± 0.27 | | 0.98 ± 0.42 | 0.80 ± 0.41 | 1.02 ± 0.58 | 0.76 ± 0.61 | 0.85 ± 0.40 | 0.93 ± 0.38 | 0.91 ± 0.44 |
| NRF2 | 1.00 ± 0.24 | | 0.94 ± 0.31 | 0.98 ± 0.39 | 0.89 ± 0.34 | 1.29 ± 0.65 | 0.82 ± 0.35 | 1.29 ± 0.78 | 0.82 ± 0.40 |
| PPARδ | 1.00 ± 0.63 | | 0.68 ± 0.57 | 0.72 ± 0.55 | 0.57 ± 0.35^a^ | 0.92 ± 1.01 | 0.50 ± 0.32 | 1.03 ± 0.79 | 0.72 ± 0.49 |
| PPARα | 1.00 ± 0.36 | | 1.04 ± 0.19 | 0.94 ± 0.18 | 0.97 ± 0.17 | 1.05 ± 0.30 | 0.95 ± 0.14 | 1.05 ± 0.27 | 1.00 ± 0.28 |

^a^There was 1 fewer sample from this treatment group

| **Table S4.** Mean enzyme activity relative to soluble protein content (mg/mL) of each antioxidant enzyme in the liver and pectoralis muscle. The linear models in Table 4 include Julian date as a covariate (n=2 or 3 per date), however mean enzyme activity (units for CAT and GPx, *nmol/min/mg* and SOD, *U/mg)* ± standard deviation are reported for each treatment group, and treatment groups are abbreviated as follows: 13 or 32% PUFA, Unsupplemented (U) or Supplemented (S) with antioxidants, and Untrained (UT) or Flight-Trained (T) for training. The number of individuals for each treatment groups are reported in the first row for the liver, pectoralis. | | | | | | | | | | |  |
| --- | --- | --- | --- | --- | --- | --- | --- | --- | --- | --- | --- |
|  | | | *Mean Antioxidant Enzyme Activity* ± *Standard deviation*  *by Diet and Flight Training Treatment Groups in the Liver and Pectoralis:* | | | | | | | |  |
|  | | | 13U, UT | | 13U, T | 13S, UT | 13S, T | 32U, UT | 32U, T | 32S, UT | 32S, T |
| *No. Individuals: Liver, Pect.* | | | *10, 10* | | *13, 13* | *10, 10* | *13, 13* | *10, 10* | *11, 12* | 8, 10 | *12, 13* |
|  | CAT | | 62.16 ± 28.7 | | 53.34 ± 19.8 | 71.33 ± 15.8 | 43.69 ± 21.1 | 74.93 ± 23.8 | 47.06 ± 20.2 | 61.85 ± 27.9 | 47.26 ± 26.4 |
| ***Liver*** | | SOD | | 1.48 ± 0.76 | 1.74 ± 0.34 | 1.84 ± 0.19 | 1.60 ± 0.50 | 1.73 ± 0.35 | 1.63 ± 0.45 | 1.58 ± 0.52 | 1.56 ± 0.57 |
|  | GPx | | 12.41 ± 7.8 | | 9.75 ± 3.6 | 11.57 ± 3.9 | 9.82 ± 4.8 | 12.91 ± 4.6 | 8.93 ± 3.7 | 12.00 ± 5.7 | 9.15 ± 3.7 |
|  | CAT | | 1.12 ± 0.35 | | 0.85 ± 0.34 | 1.24 ± 0.21 | 0.75 ± 0.41 | 0.91 ± 0.45 | 0.78 ± 0.34 | 1.31 ± 0.25 | 0.73 ± 0.32 |
| ***Pectoralis*** | SOD | | 6.64 ± 1.1 | | 6.94 ± 0.96 | 7.18 ± 0.54 | 6.83 ± 1.2 | 6.86 ± 1.1 | 7.18 ± 0.84 | 6.50 ± 0.64 | 7.53 ± 0.83 |
|  | GPx | | 7.88 ± 2.1 | | 6.91 ± 1.1 | 7.35 ± 1.5 | 7.61 ± 1.3 | 7.31 ± 1.3 | 7.36 ± 1.1 | 7.01 ± 1.4 | 7.62 ± 1.3 |
